# Paediatric Primary Care Across Europe: A Survey of 42 Countries

**DOI:** 10.1111/apa.70404

**Published:** 2025-12-04

**Authors:** Nora Karara, Antonio Corsello, Stefano del Torso, Adamos Hadjipanayis, Christine Magendie, Diego van Esso, Zachi Grossman, the European Academy of Paediatrics and the European Confederation of Primary Care Paediatricians

**Affiliations:** ^1^ European Academy of Paediatrics Brussels Belgium; ^2^ Universitätsklinikum Ruppin‐Brandenburg Neuruppin Germany; ^3^ Charité Universitätsmedizin Berlin Berlin Germany; ^4^ University of Milan Milan Italy; ^5^ University of Pavia Pavia Italy; ^6^ ChildCare WorldWide CCWW Italia OdV Padua Italy; ^7^ Medical School, European University Cyprus Nicosia Cyprus; ^8^ Pediatric Department Larnaca General Hospital Larnaca Cyprus; ^9^ Association Française de Pédiatrie Ambulatoire Ancenis France; ^10^ European Confederation of Primary Care Pediatricians Lyon France; ^11^ GRINDOPE Barcelona Spain; ^12^ Department of Pediatrics, Adelson School of Medicine Ariel University Pediatrics Ariel Israel; ^13^ Department of Pediatrics Maccabi Health Care Services Pediatrics Tel Aviv Israel

**Keywords:** adolescent health, child health, children, digital health, electronic health records, health workforce, nurses, paediatric primary care

## Abstract

**Aim:**

This study provides an updated overview of primary care for children and adolescents across Europe, investigating how systems are evolving and identifying progress and persistent gaps in healthcare models, workforce challenges and digital health integration. This study draws implications for policy makers to improve the quality of paediatric care in Europe.

**Methods:**

A cross‐sectional survey was conducted among national delegates of the European Academy of Paediatrics and the European Confederation of Primary Care Paediatricians. Respondents from 42 member countries provided standardised, national‐level data on real‐world implementation.

**Results:**

Paediatrician‐led, general practitioner‐led, and combined models were reported in 28.6%, 26.2% and 45.2% of 42 countries, respectively. Eight countries (28.6%) changed their delivery models between 2010 and 2024, most transitioning toward combined care. Paediatricians' involvement declined with patient age, while general practitioners' involvement increased. Only 35.9% of countries achieved fully interoperable electronic health records. Preventive care delivery, adolescent health visits and training duration in primary paediatric care revealed critical gaps.

**Conclusions:**

Despite progress in care integration and digital uptake, major disparities remain for adolescents, digital infrastructure and training quality. Future paediatric primary care models must harmonise training, address regional disparities and invest in interoperable digital health systems to ensure equitable, high‐quality care.

## Summary

- This first comprehensive reassessment of European paediatric primary care since 2010 captures major care delivery, workforce and digital health transformations across 42 countries.
- Twenty‐eight percent of countries restructured toward combined‐care models; critical gaps persist in training standards, digital interoperability and adolescent care
- Harmonised training, optimised digital infrastructure and adolescent care strategies are essential policy priorities for equitable, high‐quality paediatric primary care across Europe.

**Abbreviations**
EAP: European Academy of Paediatrics
ECPCP: European Confederation of Primary Care Paediatricians
EHR: electronic health record
GP/FD: general practitioner/family doctor
PCP: primary care paediatrician
PPC: paediatric primary care

## 1 Introduction

Primary care for paediatric patients is essential for high‐quality child health outcomes and system efficiency. As scientific organisations, the ‘European Academy of Paediatrics’ (EAP), the official Paediatric Section of the European Union of Medical Specialists (UEMS) and the ‘European Confederation of Primary Care Paediatricians’ (ECPCP) are dedicated to improving paediatric care in Europe [1]. Founded in 1961, the EAP focuses on promoting high‐quality care through education, training and research. EAP's membership spans the WHO European Region and Western Asia. ECPCP, established in 2009, advocates for the recognition of primary care paediatricians and advancement in comprehensive, community‐based care. Together, these organisations aim to harmonise paediatric standards and champion the health and rights of European children and adolescents. This study was initiated to provide an updated overview of how paediatric primary care (PPC) systems are currently implemented in the European region.

PPC system topology—including primary care paediatricians (PCPs), general practitioners/family doctors (GPs/FDs) or a combination of both—are shaped by historical, political and workforce factors and provide a comparative framework for analysing national healthcare systems.

Katz et al. suggested that GPs often filled rural care gaps due to economically constrained and dispersed populations, based on their survey of 34 countries [2]. In an EAP study from 2010, a paediatrician‐based model was present in 7 of 29 countries (24%) typically in Southern Europe, 10 countries (35%) reported combined/mixed models and 12 (41%) relied on GP/FD models common in Northern Europe [3]. Concerns were raised about unstandardized training in paediatric care and recommendations made for a common trunk in paediatric postgraduate training [4, 5]. A decade ago, the sustainability of an aging European paediatric workforce was flagged, yet retention struggles in ‘medical deserts’ remain very acute concerns today [3, 6].

Digital health technologies, such as electronic health records (EHRs), telemedicine and data‐sharing systems have grown, with the COVID‐19 pandemic and current conflicts and crises further underscoring the critical role of digital infrastructure in coordinated and effective care [7, 8, 9, 10]. Considering Europe's current estimated workforce shortage, digital demands, complex adolescent health needs and rising healthcare costs, reassessment is particular timely [11, 12, 13]. Fragmented monitoring and inconsistent implementation of child health strategies continue to hinder cross‐country comparison and progress toward Sustainable Development Goal 3 (SDG 3) on health and well‐being [14, 15, 16].

Europe's diversity, including significant population movement and refugee children displaced by conflict, poses both challenges and opportunities for primary healthcare providers, systems and policymakers. Culturally competent care bridging linguistic and social divides is becoming increasingly essential for delivering effective medicine [17]. Strengthening PPC is therefore more urgent than ever, as early‐onset noncommunicable and mental health conditions now contribute substantially to the burden of disease (DALYs) in children and adolescents across Europe [18].

This study provides a comprehensive, descriptive overview of PPC structures in 42 European and neighbouring countries, establishing an actual evidence base for policymakers and health authorities.

## 2 Methods

This cross‐sectional study used a questionnaire adapted from previous surveys by Katz et al. and van Esso et al., covering PPC systems, demographic data, paediatric visits, vaccinations and postgraduate training with updated content addressing adolescent care and digital infrastructure [2, 3]. The questionnaire (Table S1) included explanatory text for each multiple‐choice or multiple‐select question to ensure consistent interpretation across diverse health systems. We did not include open text fields.

The survey was distributed electronically via the EAP Research in Ambulatory Settings network (EAPRASnet) platform between May and June 2024 to national delegates of the EAP and ECPCP, representing 45 countries [19]. Delegates, nominated by their national paediatric societies, included both primary care and hospital‐based paediatricians with a solid understanding of primary paediatric care. Respondents were instructed to describe the predominant national system to enable systematic cross‐country comparison, while acknowledging that regional variations may exist within countries.

We employed a national informant approach to gather information on country‐level healthcare structures. This follows established precedent in European health systems research including the Models of Child Health Appraised (MOCHA) study [20]. To obtain a single representative response to each item, discrepancies were resolved by consulting an additional national paediatric expert. If no consensus could be reached, the corresponding item was excluded from the analysis. Additionally, responses marked as ‘not applicable’ were excluded from the denominator for that item to avoid misrepresenting the prevalence of system‐level features.

For comparability with previous research, we replicated the system classification analysis for the same 29 countries included in the van Esso et al. survey [3] before extending to all 42 participating countries. The descriptive analysis was conducted using Microsoft Excel.

## 3 Results

Forty‐two of 45 invited countries participated (93.3%), with 77 individual responses from 121 contacted EAP and ECPCP national delegates (63.6%). Figure S1 shows respondents per country.

### 3.1 Paediatric Primary Care Systems and Access to Care

**FIGURE 1:**
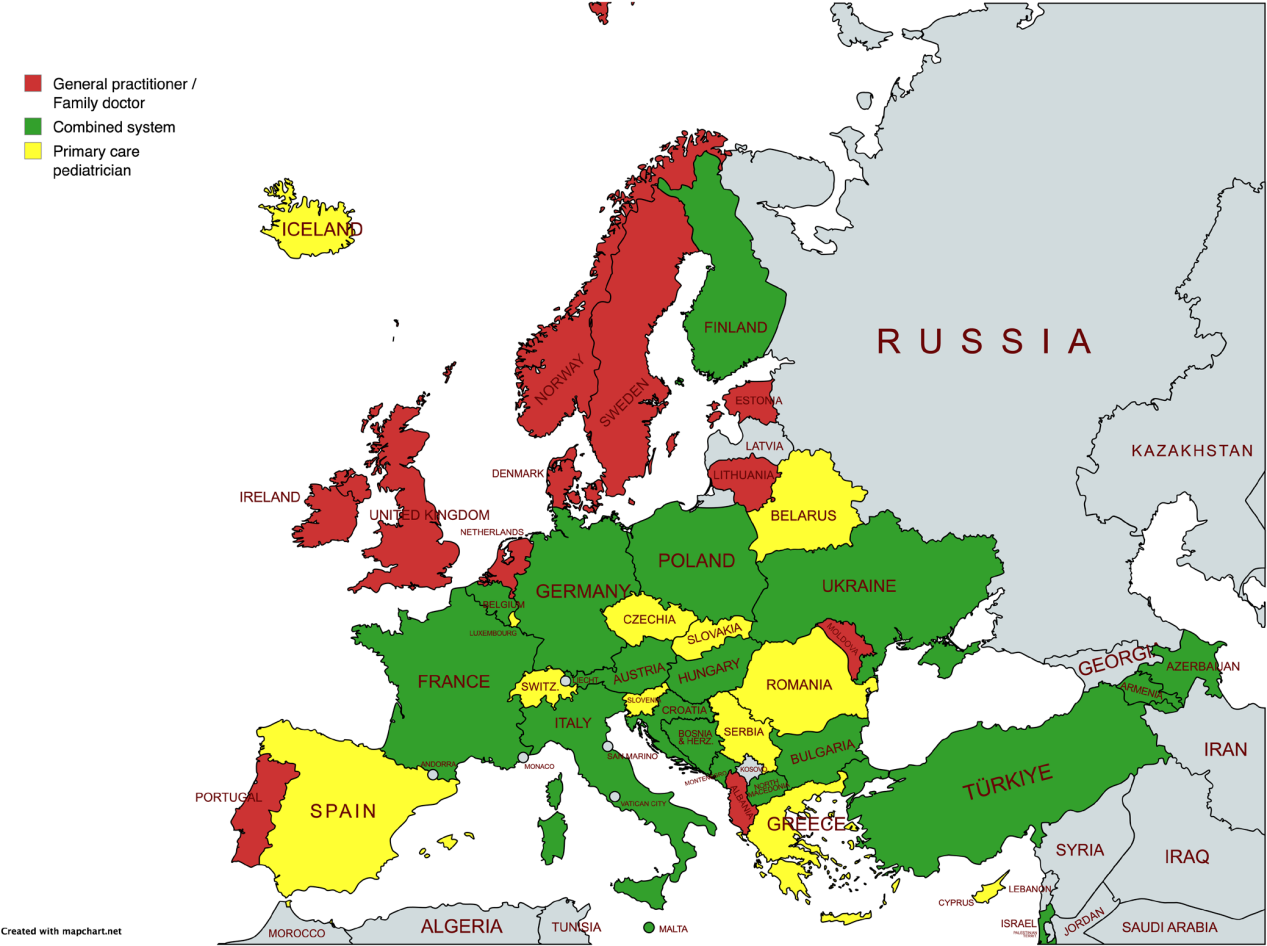
PPC System Structures in Europe 2024. Map represents national primary care system structures as reported by national paediatric experts. Local delivery patterns may vary.

In the full 42‐country dataset, combined systems were most common (19 countries, 45.2%). Paediatrician‐based systems were reported in 12 countries (28.6%), GP/FD systems in 11 countries (26.2%). The current geographic distribution of PPC in Europe is shown in Figure 1. For comparison with 2010 see Figure S2.

Compared to the 28 countries from van Esso et al. in 2010, eight countries (28.6%) had restructured their PPC delivery models: Bulgaria, Finland and Poland transitioned from GP/FD‐based systems to combined models; Israel from a paediatrician‐based to a combined system. Iceland, Luxembourg and Switzerland transitioned from a combined to a paediatrician‐based system, while Lithuania moved toward a GP/FD system. Additionally, in consensus finding, Greece and Cyprus reported a paediatrician‐based system but noted increased GP/FD involvement, while the Netherlands described a distinct separation between preventive and acute care delivery.

In terms of health insurance, the majority (30/42 countries, 71.4%) had public insurance or a national health system. Mixed private‐public insurance systems were present in 10 countries (23.8%). Romania was the only Eastern European country with a mixed insurance system. Private insurance was only reported in 2 countries (4.8%): Netherlands and Switzerland. Most countries (35/42, 83.3%) offered free access to PPC, while the remaining 7 countries required either copayment (Belgium, Iceland, Spain) or payment with reimbursement options (Greece, France, Luxembourg and Switzerland).

### 3.2 Provider Involvement Across Age Groups

**FIGURE 2:**
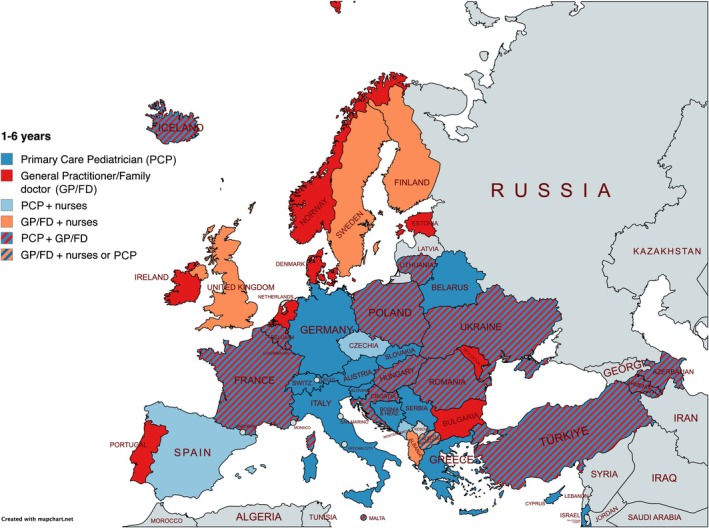
PPC providers for children aged 1–6 years old.

**FIGURE 3:**
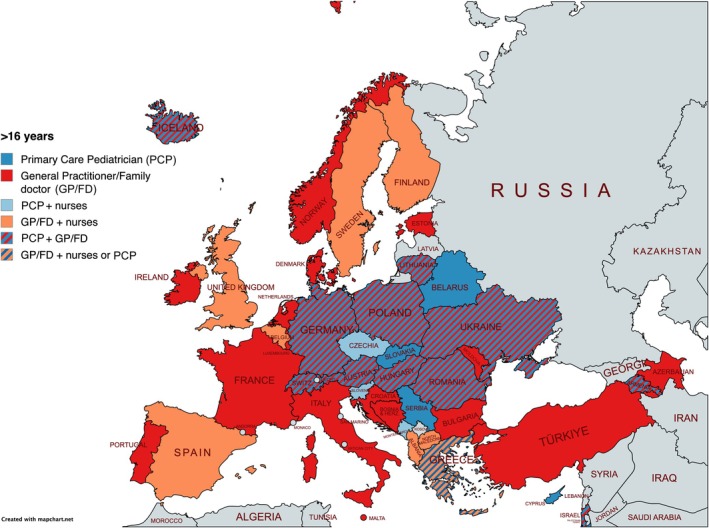
PPC providers for children aged ±16 years old.

Figures 2 and 3 illustrate provider involvement by age group across countries. As respondents could select multiple provider types per age range, these reflect shared responsibilities. Additional age‐specific maps and a summary graph are presented in Figures [Link], [Link], [Link], [Link], [Link].

**FIGURE 4:**
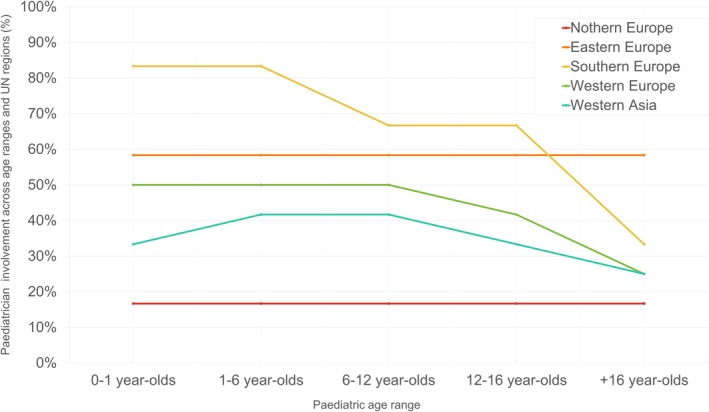
Paediatrician involvement in different age ranges across UN Regions (compare Table S2).

Paediatricians were reported as providers in 29 of 42 countries (69.0%) for children aged 0–1 years and 30 countries (71.4%) for those aged 1–6 years. Involvement gradually decreased with age: 28 countries (66.7%) for 6–12 years, 26 (61.9%) for adolescents aged 12–16 years. The least involvement of paediatricians was reported for adolescents aged 16 years and older by 19 of 42 countries (45.2%), reflecting 10 countries reporting less involvement, a decrease of 23.8% from early childhood to late adolescence. Regionally, this decline was most pronounced in Western and Southern Europe after age 12 (Figure 4).

In contrast, GP/FDs were reported in 24 of 42 countries (57.1%) for the 0–1‐year‐old group, with involvement increasing by 12 reporting countries (+28.6 percentage points) as children grew older: 32 countries (76.2%) for ages 6–12 and rising to 36 countries (85.7%) involving GPs/FDs for adolescents aged 16 and older.

**FIGURE 5:**
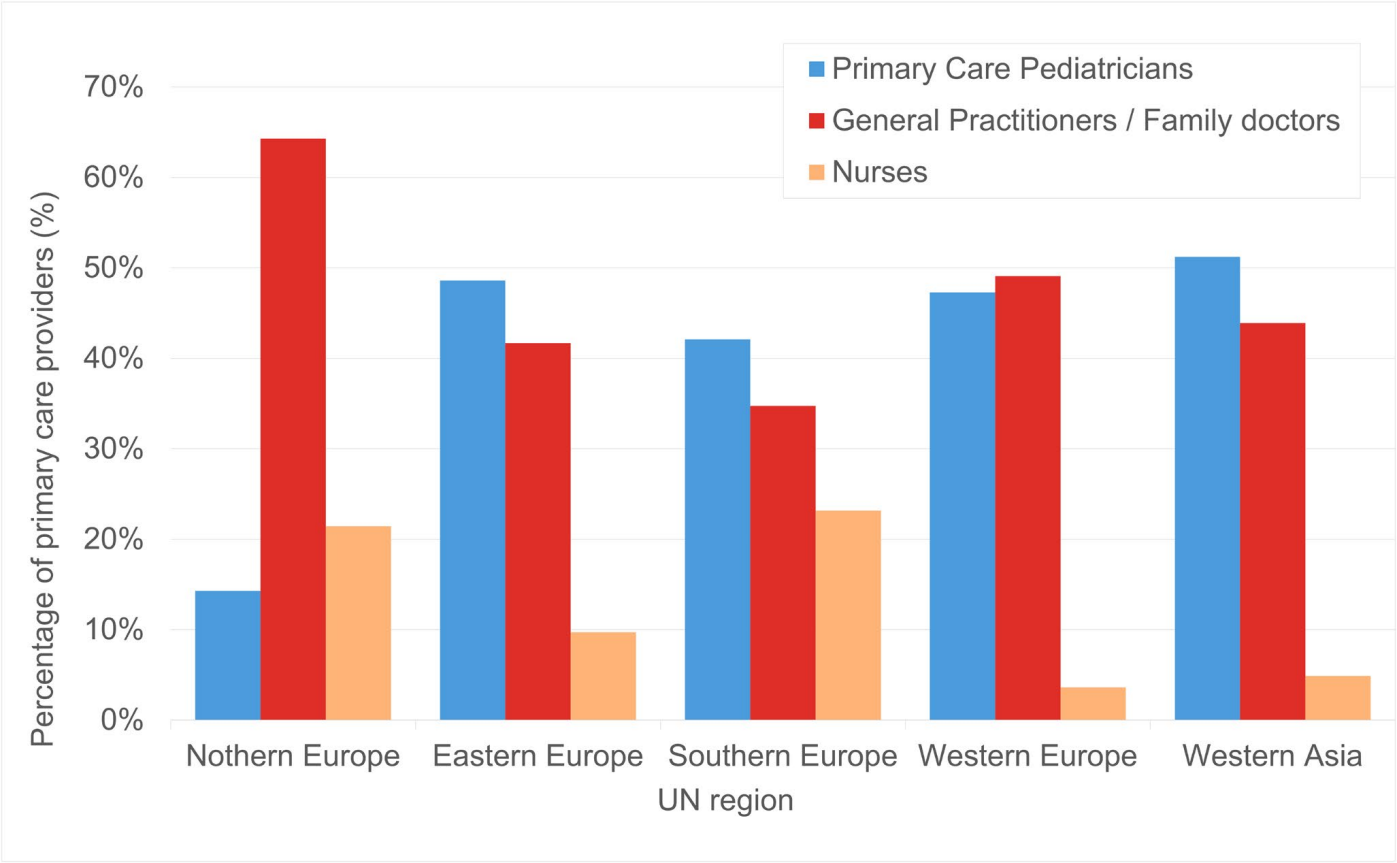
PPC providers' involvement across UN regions (compare Table S2).

Nurses were reported as primary providers of PPC in 13 of 42 countries (31%) for the 0–1‐year age range, their highest reported proportion across age groups. Results of our multiple select question for regional patterns are shown in Figure 5.

Primary care settings included 10 of 40 countries (25%) relying on paediatricians' offices. A multidisciplinary approach with clinics or health centers involving nurses, social workers and other health professionals was present in 24 of 39 countries (61.5%), with 11 countries indicating these interdisciplinary health centers as the only place where children receive primary care. Compared to 2010, this represents a 25.5 percentage point increase in multidisciplinary team involvement over the 14‐year period.

### 3.3 Training

Training duration varied considerably across countries and provider types. Specific training for paediatricians in PPC was very short with 18 of 37 countries (48.6%) requiring a minimum training period of under 7 months.

Regarding GP/FD training in Paediatrics, data from 38 countries was available. 21 countries (55.3%) reported a training duration of 1–3 months while 12 (31.6%) indicated 4–6 months. No required paediatric training for GPs/FDs was reported in five countries (13.1%), notably occurring in three GP/FD‐led systems (Netherlands, Norway, UK). Four countries (Azerbaijan, Belarus, Luxemburg and Slovakia) marked the question as not applicable.

### 3.4 Preventive Care Delivery: Health Visits and Vaccinations

Twenty‐nine of 42 countries (69.0%) scheduled more than nine routine visits, whereas 4 countries (9.5%) scheduled only 1–3 visits (Azerbaijan, Bosnia Herzegovina, Iceland, Romania). Adolescent health revealed vast contrasts in preventive care, as 25 countries (59.5%) offered at least two routine visits, but in 6 countries (14.3%)– —Austria, Cyprus, Israel, Luxemburg, Malta, Norway‐ adolescents were offered none.

Regarding the professionals in charge of these routine child well‐visits, PCPs led care in 24 of 41 reporting countries (58.5%) with 21 of 24 indicating more than 9 visits. GP/FDs were responsible in 11 of 41 countries (26.8%), which showed high variation in visit frequency ranging from 1 to 3 up to more than 9 visits. Specialised nurses led preventive care in 6 of 41 countries (14.6%), primarily in Northern Europe (Finland, Norway, Sweden, UK) and Israel, which reported moderately frequent visits.

Childhood vaccinations delivery utilised multiple settings in 29/42 countries (69.0%). Single‐setting delivery occurred via paediatrician offices in 4 countries (Cyprus, Czechia, Slovakia and Slovenia), GP/FD offices in 3 countries (Bulgaria, Denmark, Romania) and public health centers in 5 countries including the Netherlands, Lithuania and Portugal.

Adolescent vaccination prominently occurred in school‐based programs (17/41 countries, 40.5%), with Norway and Israel using this setting exclusively. Sweden uniquely reported school‐based delivery for all children and adolescents of school age.

### 3.5 Digital Health and Telemedicine

Among 42 reporting countries, 20 (47.6%) had established digital health tools, 19 (45.2%) were gradually introducing them. Three countries (7.1%) not routinely using EHRs were excluded from related analysis, and several could reach ‘no consensus’ for certain EHR indicators due to regional differences.

EHRs including medical history were maintained in all 38 valid responding countries. Digital prescriptions were nearly universally used (37/38 countries, 97.4%), followed by lab tests/blood work with 35 of 36 countries (97.2%) and electronic vaccination records used in 36 of 39 countries (92.3%). Digital recording of child well visits information such as milestones saw less implementation with 30 of 36 countries (83.3%) as well as imaging data on EHRs present only in 28 of 36 countries (77.7%).

**FIGURE 6:**
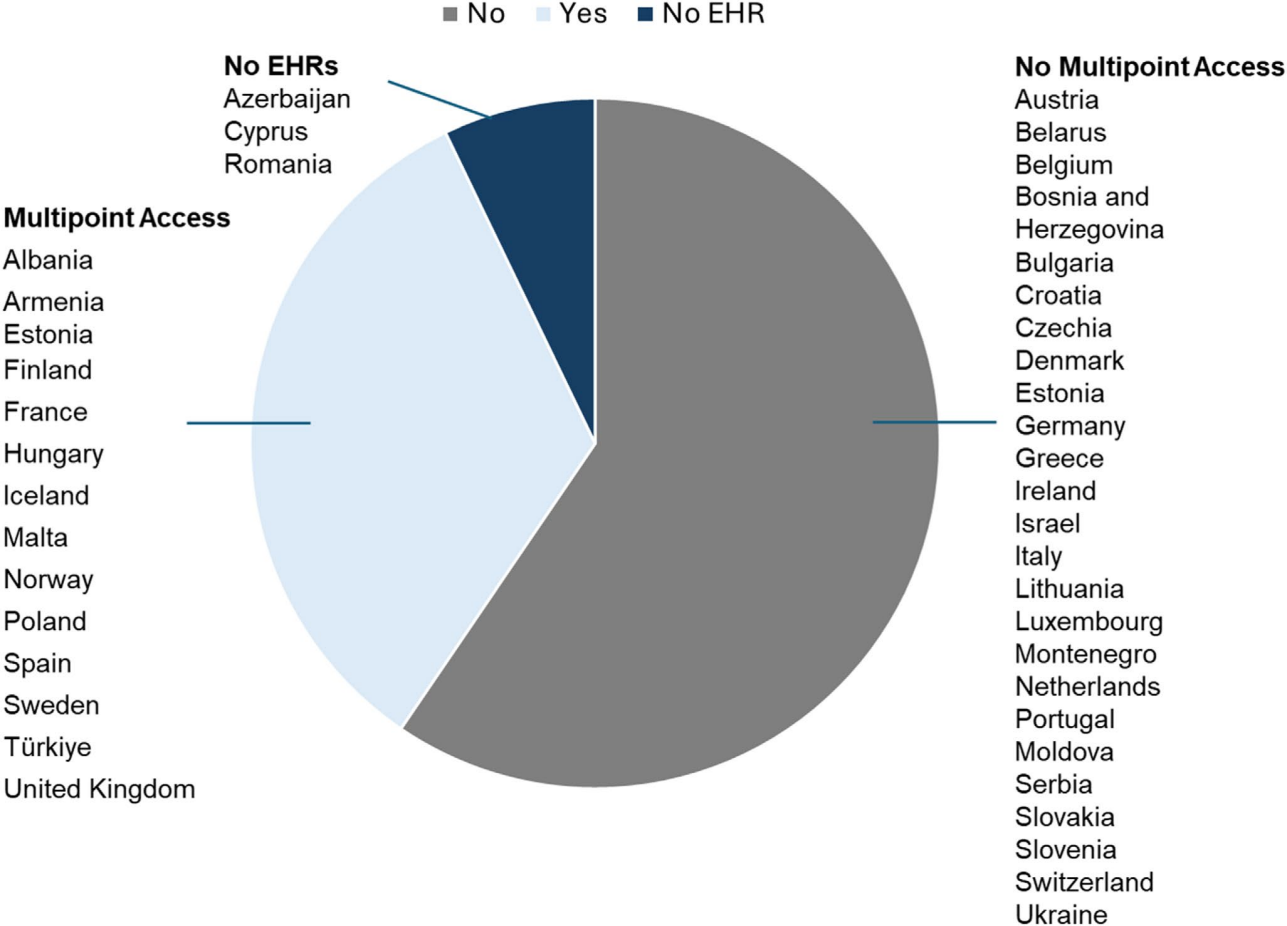
Distribution of multiple access points to electronic health records.

The utility of these EHR features depends on accessibility across healthcare settings. Access to EHRs was granted firstly to PCPs/GPs (36 of 38 responding countries, 94.7%). Pharmacies had access in 30 of 35 (85.7%), hospitals in 29 of 38 countries (76.3%) and only 29 of 37 responding countries (78.4%) offered patients access to their EHRs. Notably just 14 of 39 countries (35.9%) indicated the availability of multiple access points, representing 33% of all 42 countries surveyed (see Figure 6).

Only 6 countries—Finland, Hungary, Iceland, Norway, Sweden from Northern Europe and Türkiye from Western Asia—combined full implementation across all EHR features with multipoint access capabilities. Two countries (France and the United Kingdom) reported all assessed EHR features including multipoint access yet remained in gradual implementation phases. Eleven countries—Belgium, Croatia, Denmark, Israel, Lithuania, Malta, Montenegro, Netherlands, Portugal, Slovenia and Ukraine—reported established EHR systems with multiple features but lacked multipoint access capabilities. Seven countries (Bosnia and Herzegovina, Bulgaria, Germany, Ireland, Luxembourg, Serbia and Switzerland) reported the combination of limited EHR features, no multipoint access and gradual implementation status.

Regarding telemedicine adoption, several countries reported regional disparities for specific services, leading to exclusion from those particular analyses. Telemedicine was used primarily for chronic disease management (20/35 countries, 57.1%). Use for acute care was less frequent (14/33, 42.4%). Mental health consultations via telemedicine were available in only 9 of 30 countries (30%). Ten of 33 countries (30%) reported no paediatric telemedicine use whatsoever.

## 4 Discussion

EAP in 2010 predicted that without urgent attention to workforce planning, paediatric primary care systems might be forced to undergo fundamental restructuring [3, 19]. A decade later, this study documents that transformation: eight of 28 comparable countries (28.6%) modified their delivery models. Most transitioned toward combined models (Bulgaria, Finland, Poland and Israel) with notable exceptions moving to specialised approaches. Nearly half (45.2%) of all countries surveyed in the full 42‐country dataset now operate combined systems. With a 93.3% response rate, this study provides comprehensive insights reflecting workforce pressures and changing care demands across the European regions.

### 4.1 System Evolution, Workforce and Care Delivery

Leveraging the strengths of both PCPs and GPs with improved cooperation can optimise workforce distribution and enhance access to care, particularly in underserved areas [11, 21]. However, our data reveal a critical disconnect in provider preparedness: 29 of 42 countries (69.0%) involve GPs/FDs in care from infant age onwards, yet 21 of 38 countries (55.3%) reported minimal required training duration in Paediatrics of only 1–3 months for GPs/FDs. Five countries (Italy, the Netherlands, Norway, Switzerland and the United Kingdom) reported that no paediatric training was required for GPs/FDs at all. While an early move to a well‐trained GP/FD might lead to an easier transition to adult care, proper standards, especially for adolescents with complex care needs, are still lacking [22]. Among paediatricians, training required in primary care was similarly limited, with 18 of 37 countries (48.6%) requiring fewer than 11 months.

While upholding a resilient healthcare workforce with specialised paediatric knowledge is vital to ensure child and adolescent health, especially after the COVID‐19 pandemic, [23] these variations represent an ongoing systems gap, potentially compromising early detection of developmental conditions and the provision of age‐appropriate preventive care [21, 24].

### 4.2 Preventive Care Delivery Models

European PPC systems are also recalibrating through internal structural changes. Multidisciplinary team care (e.g., including physiotherapists, dietitians, psychologists) increased from 9 of 25 countries (36%) in 2010 to 24/40 (60%), exemplifying the WHO Framework on Integrated, People‐Centred Health Services (IPCHS) and suggesting both preventive services and management of chronic conditions within primary care models [20, 25]. This evolution may reflect rising case complexity, efforts to improve care quality through diverse professional expertise, and potential strategic resource sharing amid increasing costs [24, 26].

Our findings demonstrate substantial heterogeneity in preventive care frequency and delivery setting, representing missed intervention opportunities during key developmental periods. Four of 42 countries (Azerbaijan, Bosnia Herzegovina, Iceland and Romania) offered only 1–3 health visits to children. Variation appears linked to system type more than region: GP‐led systems reported the most variation in routine visits (1–3 to > 9), across Southern Europe (Albania, Bulgaria and Portugal), Northern Europe (i.e., Iceland, Denmark) and Eastern Europe (Romania, Moldova), potentially prioritising episodic over structured well‐child programs. In contrast, paediatricians‐led systems (Italy, France and Germany) reported consistently more frequent visits. Future research should evaluate health outcomes such as vaccination coverage and access to essential services associated with care delivery models. Our age‐specific provider analysis reveals an important finding that system classifications may obscure: paediatricians predominantly care for younger children while GPs/FDs assume greater responsibilities as children age.

### 4.3 The Adolescent Care Gap

The most concerning pattern emerges in adolescent care. While promising that over half of the countries offered at least two visits for adolescents, six of 42 countries (14%) scheduled none and 11 countries (26.1%) only one. This gap is compounded by a provider paradox: paediatrician involvement decreased by 23.8 percentage points from infancy to late adolescence, while GP/FD responsibilities increased by 28.6%. Thus, the most complex age group receives care from the least paediatric‐specifically trained. Adolescence is a vital stage for supportive, age‐appropriate guidance with lasting impact on future well‐being, making specialised PPC training crucial [27, 28].

### 4.4 Training

While combined systems may offer advantages through improved workforce distribution and care access, their effectiveness depends critically on provider preparation. The substantial variation in training requirements suggests that harmonised training standards remain underdeveloped despite progress in the creation of European Training Requirements (ETRs) in paediatric disciplines [29].

Establishing core competencies—not uniformity, but quality standards—would ensure all providers possess adequate preparation. National strategies incentivising specialised training through financial support and career development represent essential steps toward sustainable workforce solutions. Without harmonised standards, European paediatric health systems risk maintaining access while compromising provider competency across diverse clinical scenarios [28].

### 4.5 Coordinated Care Models

Developing European standards for coordinated care models represents a strategic opportunity to ensure comprehensive PPC across all age groups [16]. The ‘medical home’ model, as endorsed by the American Academy of Paediatrics [30] emphasises integrating preventive, primary and specialty care. However, standardised protocols for routine health visits remain scarce, with limited evidence on optimal visit frequency or procedures, requiring further in‐depth research to generate robust evidence across diverse European health contexts.

### 4.6 Digital Health

The European Health Data Space (EHDS) regulation mandates interoperable health data systems across EU member states by 2025 [31], yet our findings reveal a stark implementation deficit in regard to Paediatrics. Nineteen countries (45.2%) are still gradually introducing EHRs and, more critically, only fourteen countries (38.8%) reported multipoint access to EHRs allowing data sharing across primary care, hospitals, pharmacies and patients. Twenty‐two countries (61.1%) do not or cannot share paediatric healthcare data strategically even within their own healthcare systems, effectively isolating paediatric health data within institutional silos. Although some regions within larger, decentralised countries may have achieved greater interoperability, our national‐level assessment captures the predominant system accessibility experienced by most children, revealing implementation gaps that echo findings from Grossman et al. [7].

Only 6 countries—mostly in Northern Europe—combine extensive EHR features with multipoint access, gaining capacity to actually use EHRs in paediatric primary care to full advantage. These disparities undermine broader European digital health initiatives. A critical barrier is that most digital health systems are designed without paediatricians' input or specific workflow considerations, leading to systems that fail to capture child‐specific data—evident in the gaps shown in the milestone chart feature in our study. Blind spots like these contribute to children's invisibility in digital health monitoring, create operational inefficiency for PPC and hamper evidence‐based policymaking [16].

Beyond EHRs, telemedicine adoption reveals similar patterns of digital fragmentation. Results indicated a broader regional digital divide with Eastern European countries showing polarization between digital leaders and countries with limited implementation. Fifteen of 39 countries (38.5%) reported no paediatric telemedicine adoption, missing a key component of digital health infrastructure.

Robust data sharing and health record continuity represent critical determinants of PPC health system resilience, particularly given the increasing mobility of families and especially those fleeing conflicts. While home‐based records remain widely used and can, according to WHO, support participation of parents and continuity when well maintained, their effectiveness is constrained by incomplete documentation and vulnerability to physical loss during displacement [32]. Electronic systems can offer substantial advantages for real‐time data sharing, vaccination tracking and continuity during population movement [8, 33]. However, our findings reveal only 35.9% of countries have achieved multi‐point interoperability. This widespread non‐compliance with pending EU requirements highlights the urgent need for coordinated policy interventions and technical support.

As healthcare systems worldwide move toward digital integration, interoperable electronic health systems represent the essential infrastructure for 21st‐century paediatric primary care. Standardised formats, training and integrated systems are necessary to ensure health professionals and families can utilise paediatric health information effectively and consistently across borders [34]. To realise digital health's full transformative potential, European countries must prioritise three actions: expand multipoint EHR access, ensure robust data governance frameworks and invest in paediatric‐specific digital health capabilities.

### 4.7 Access and Health Equity

Healthcare equity challenges persist despite demonstrated system evolution. Post COVID‐19 data reveal 5% of low‐income families with children under 16 experienced unmet medical needs, with the highest levels found in towns and suburbs across Romania and Hungary [35]. While most countries offer free access to PPC, hidden additional costs such as medication provisions and informal payments, create additional barriers, in particular for vulnerable populations [36].

Addressing these barriers requires multifaceted approaches. Digital innovations offer promising solutions for reaching underserved populations, including children made vulnerable by migration who face language and cultural barriers in traditional PPC settings [17, 37]. However, achieving balance between innovation, and privacy remains challenging, and efforts to digitise healthcare systems may face resistance due to unclear payment practices in some settings. Healthcare systems must adopt transparent data practices and provide healthcare professionals with ethical training on digital tools in Paediatrics to maximise benefits while safeguarding trust. Contemporary socio‐political challenges to diversity and cultural competence in some European settings may further complicate efforts to provide equitable care.

### 4.8 Vaccinations and School Health

School health programs represent a critical yet underutilised opportunity for equity advancement [3, 38]. Despite WHO endorsement of health‐promoting school programs, only nine of 39 countries (23.1%) regularly administer childhood vaccinations in schools, rising to 17 of 40 countries (42.5%) for adolescents [39]. This is concerning given schools' universal reach and demonstrated effectiveness [40].

These delivery gaps reflect broader challenges in implementing comprehensive child health strategies. Despite growing recognition, not enough countries have implemented standalone child and adolescent health frameworks to ensure healthy lives and well‐being for all children [14]. Progress toward SDG 3 requires prioritising inclusive child and adolescent health as central to national agendas, particularly given the proven cost‐effectiveness of early intervention [41, 42].

PPC providers, often the first point of contact for families, play a crucial role in promoting healthy habits and addressing mental health issues during critical developmental periods.

Ensuring equitable access for all children, irrespective of their background and upholding child rights, must remain a guiding principle, requiring coordinated action beyond PPC alone [43, 44].

### 4.9 Limitations

Several methodological considerations must be acknowledged. The cross‐sectional design limits causal inferences. We focused on structural features rather than clinical processes—not capturing specific provider roles, visit content or informal payment practices. The single‐informant system and varying response rates could introduce reporting bias and mask regional differences. Inconsistent survey design complicates cross‐study comparisons (we used ‘majority’ whereas van Esso et al. [2] specified a 75% threshold). Additionally, language barriers may have affected clarity for non‐English‐speaking participants. We are currently expanding this survey globally and conducting a dedicated assessment of paediatric training structures across countries. Future work will also include differentiation of nurse types, training levels and scope of practice to better characterise multidisciplinary paediatric primary care models. Despite the limitations, this study provides a robust, expert‐informed overview from practicing paediatric professionals across 42 countries, establishing a valuable baseline for future policy development.

## 5 Conclusion

This comprehensive analysis of paediatric primary care across 42 European countries documents system transformation with 28.6% of compared countries modifying their delivery models within a decade. Three evidence‐based priority areas emerge from our findings. First, paediatric training inadequately matches assigned responsibilities: over half of the countries offer GPs/FDs just 1–3 months of training despite involvement from infancy through adolescence. Training for paediatricians lacks equal standards with no current overview of the implementation of European curricula. Second, digital infrastructure fragmentation limits care coordination, with only 38% of countries achieving multipoint EHR access. Third, preventive care delivery shows substantial variation with 14% of adolescents receiving no routine visits despite this being a critical period for intervention.

While respecting diverse national healthcare contexts, minimum quality thresholds for paediatric competencies, interoperable data systems and universal health access represent achievable policy targets that can strengthen care quality for children and adolescents regardless of geographic or socioeconomic factors, in Europe and beyond.

## Author Contributions

Nora Karara conceptualised and designed the study, collected the data, conducted the initial analyses, drafted the initial manuscript as well as critically reviewed and revised the manuscript. Antonio Corsello collected the data, conducted the initial analyses, drafted the initial manuscript and critically reviewed and revised the manuscript. Zachi Grossman conceptualised and designed the study, coordinated and supervised the data collection, critically reviewed and revised the manuscript. Stefano del Torso, Diego van Esso, Christine Magendie and Adamos Hadjipanayis conceptualised and designed the study, supervised the data collection and critically reviewed the manuscript for important intellectual content. All authors approved the final manuscript as submitted and agree to be accountable for all aspects of the work.

## Funding

The authors have nothing to report.

## Conflicts of Interest

The authors declare no conflicts of interest.

## Supporting information


**Figure S1:** survey respondents per country.

**Figure S2:** PPC system structures in 2010.

**Figure S3:** PPC providers involvement across age ranges.

**Figure S4:** Primary care providers for 0–1 years map.

**Figure S5:** Primary care providers for 6–12 years map.

**Figure S6:** Primary care providers for 12–16 years map.

**Table S1:** PPC questionnaire.

**Table S2:** Countries by geographical region according to standard country or area codes for statistical use by the UN.

## Data Availability

The data that support the findings of this study are available on request from the corresponding author. The data are not publicly available due to privacy or ethical restrictions.
