# Supplementary figures and images for: Paediatric Primary Care Across Europe: A Survey of 42 Countries
Source: Acta Paediatr. 2025 Dec 4;115(4):821–31. doi: 10.1111/apa.70404 (PMC12975683; doi:10.1111/apa.70404)

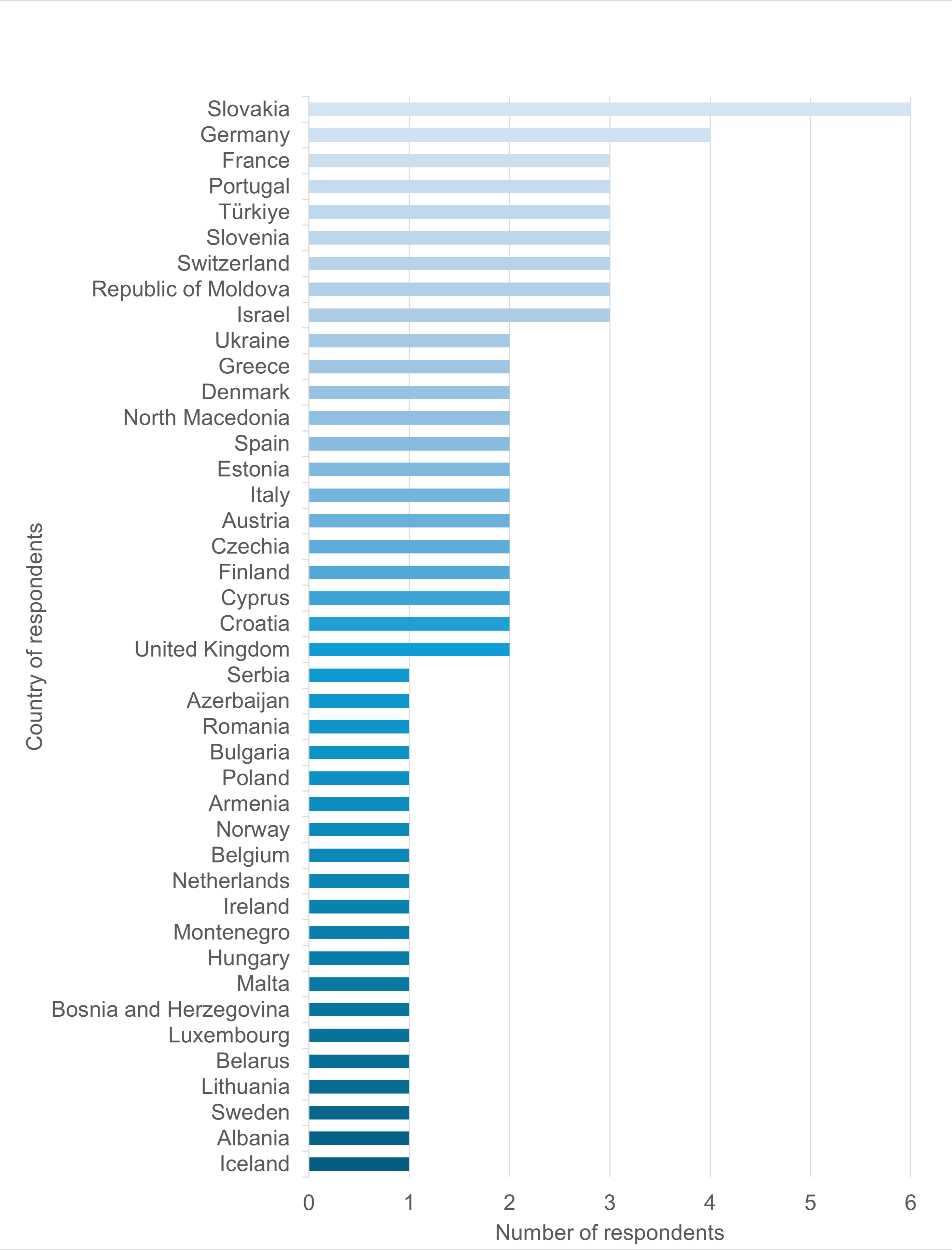

Supplement: Supplementary file 1 — Figure S1: survey respondents per country. [file APA-115-821-s006.tiff]

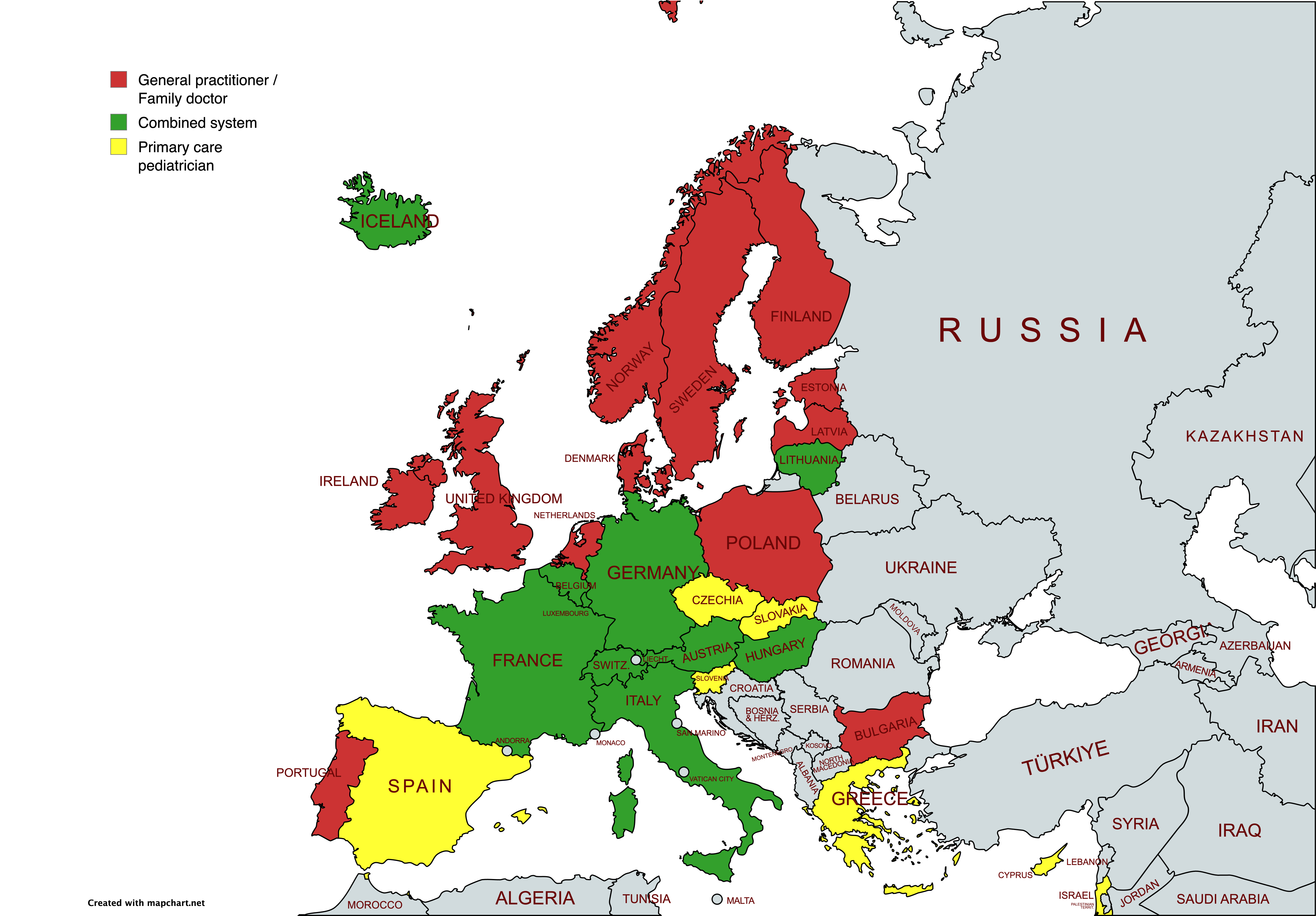

Supplement: Supplementary file 2 — Figure S2: PPC system structures in 2010. [file APA-115-821-s007.tiff]

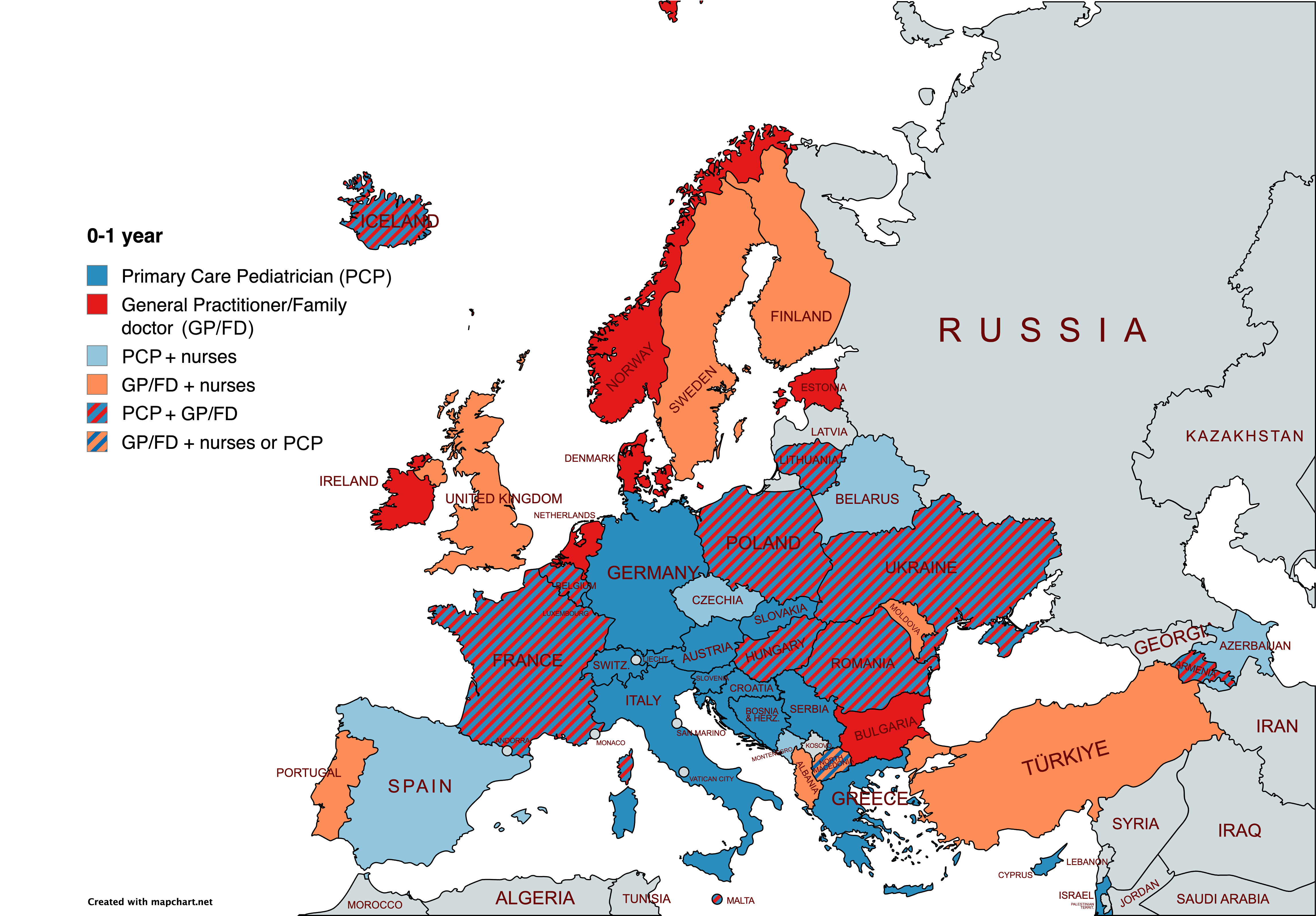

Supplement: Supplementary file 4 — Figure S4: Primary care providers for 0–1 years map. [file APA-115-821-s008.tiff]

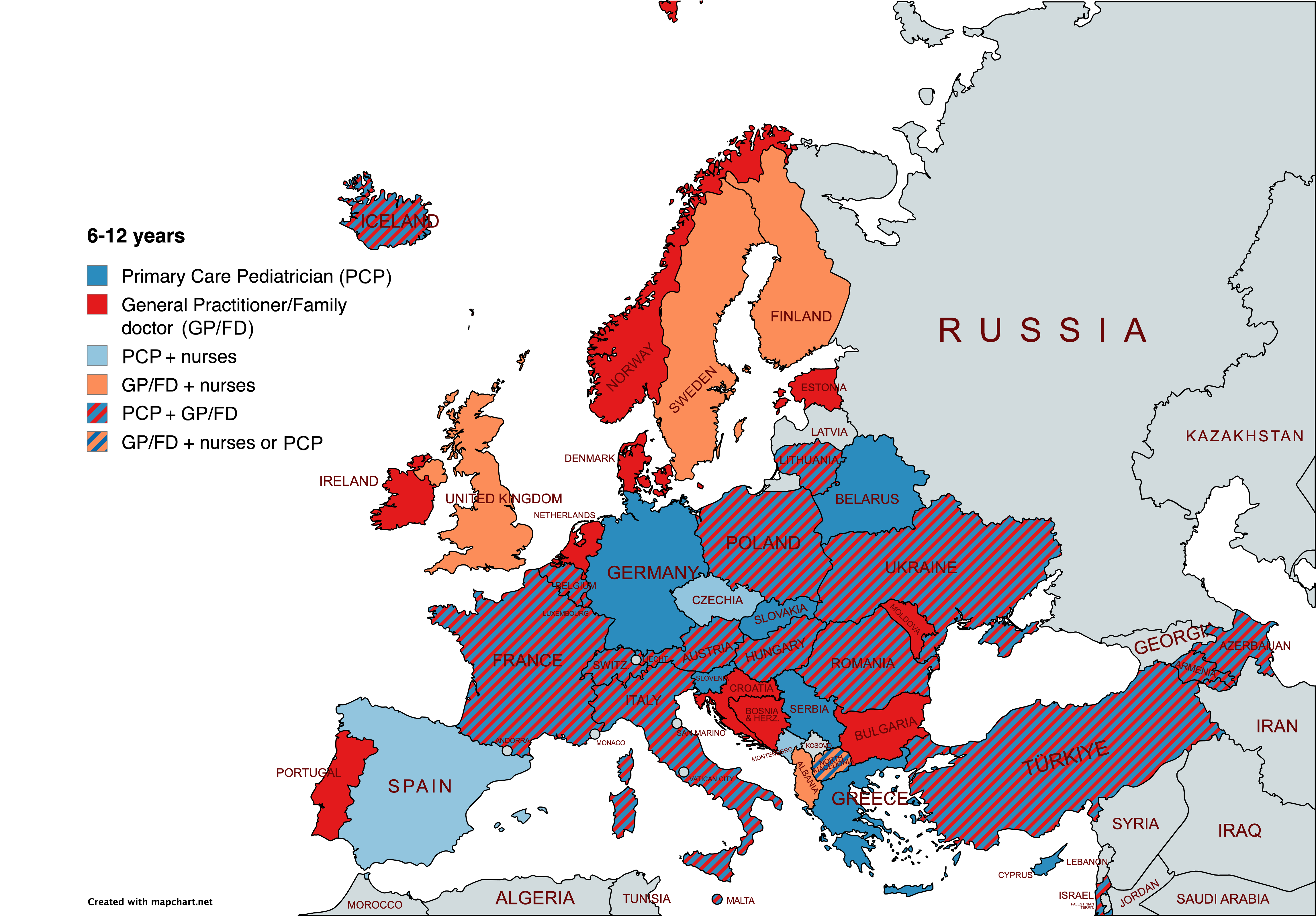

Supplement: Supplementary file 5 — Figure S5: Primary care providers for 6–12 years map. [file APA-115-821-s005.tiff]

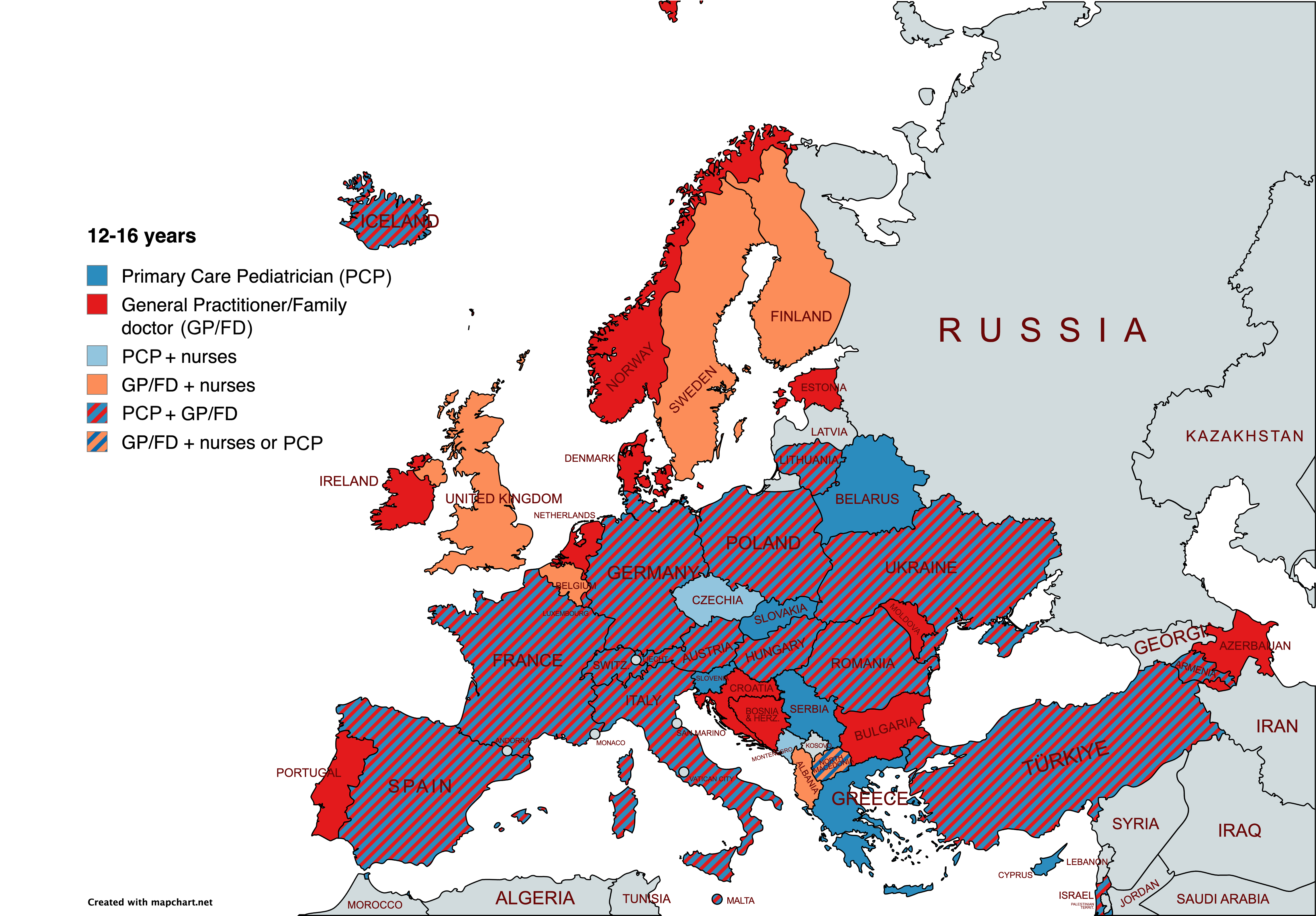

Supplement: Supplementary file 6 — Figure S6: Primary care providers for 12–16 years map. [file APA-115-821-s001.tiff]
