# Supplementary material for: Paediatric Primary Care Across Europe: A Survey of 42 Countries
Source: Acta Paediatr. 2025 Dec 4;115(4):821–31. doi: 10.1111/apa.70404 (PMC12975683; doi:10.1111/apa.70404)
Supplement: Supplementary file 7 — Table S1: PPC questionnaire. [file APA-115-821-s002.docx]

**Supplementary Tables**

***Table S1*** *Survey Questionnaire*

| **Section** | **Question** | **Response Options** |
| --- | --- | --- |
| **General Information** | 1. Country: Please provide the name of the country you work in. | [Dropdown with various countries] |
| **Type of Primary Pediatric Care System** | 2. Is your primary pediatric Care system based on general practitioners/family doctors (GP/FD), pediatricians or a combined system?  * When you answer this question, please describe the main system of your country, the system that cares for the majority of children/adolescents. Leave minor variations of the system aside | - Family doctors (FD)/general practitioners (GP)  - Pediatricians in primary care  - Combined system |
| **Insurance Type** | 3. What type of insurance system is in place for the vast majority of children in your country?  * When you answer this question, please describe the main system of your country, the system that cares for the majority of children/adolescents. Leave minor variations of the system aside | - Public (NHS)  - Private  - Mixed (Private/Public) |
| **Access to the System** | 4. How is access to the primary pediatric care system managed?  * When you answer this question, please describe the main system of your country, the system that cares for the majority of children/adolescents. Leave minor variations of the system aside | - Free (no direct payment)  - Free with a copayment  - Payment with total reimbursement  - Payment and partial reimbursement |
| **Setting and Professionals Involved in Primary Care** | 5. Who usually cares for children in different age ranges?  * When you answer this question, please describe the main system of your country, the system that cares for the majority of children/adolescents. Leave minor variations of the system aside | Select options for: |
|  | - 0-1 years: | PCP, GP/FD, Community Nurse |
|  | - 1-6 years: | PCP, GP/FD, Community Nurse |
|  | - 6-12 years: | PCP, GP/FD, Community Nurse |
|  | - 12-16 years: | PCP, GP/FD, Community Nurse |
|  | - 16+ years: | PCP, GP/FD, Community Nurse |
| **Primary Care Locations** | 6. In which place or places do children typically receive primary care? (multiple select) | - Solo pediatricians  - Group clinic of pediatricians  - Child care centres/health centers/ clinics involving nurses  - Child care centres/health centers/ clinics involving nurses, social workers, physiotherapists etc.  - Hospital setting (out-patient)  - not applicable |
| **Activities** | 7. How many routine health visits are scheduled in your national child health program? | - 1-3, 3-6, 7-9, more than 9 |
|  | 8. Who is in charge of the routine health/ child well visits?  * When you answer this question, please describe the main system of your country, the system that cares for the majority of children/adolescents. Leave minor variations of the system aside | - Primary Care Pediatrician  - Trained Nurses (Community Nurse)  - General Practitioner/Family Doctor  - not applicable |
|  | 9. How many routine adolescent health visits are scheduled in the national adolescent health program (>12years old)? | - None, 1, 2, More than 2 |
| **Vaccinations** | 10. Where are vaccinations usually administered?  * When you answer this question, please describe the main system of your country, the system that cares for the majority of children/adolescents. Leave minor variations of the system aside |  |
|  | for children: | - Pediatrician’s office  - GP/FD office  - Healthcare centers  - Schools  - not applicable |
|  | for adolescents: | - Pediatricians office  - GP/FD office  - Healthcare centres  - Schools  - not applicable |
| **Training in Pediatric Care** | 11. Is there specific training for pediatricians for PPC? | - Yes, No |
|  | 12. If yes, is it integrated during pediatric training or is it after the normal training for Pediatrics? | - Integrated, Not integrated |
|  | 13. In your country, besides the usual training in pediatric departments in hospitals, what is the minimal number in months of PCP training required for pediatricians in your country? | - None, 1-3, 4-6, 6-12 |
|  | 14. Where does training occur? | - Pediatric practice  - Outpatient Clinic/hospital  - Healthcare Centers  - GP/FD practice  - not applicable |
|  | 15. Who supervises the PPC training usually? | - GP/FD  - PCP  - Academic Pediatricians  - Trained Nurses  - not applicable |
|  | 16. Has the national residency training curricula been changed to include PPC in the last 10 years? | - Yes, No |
| **GP Training in Pediatrics** | 17. What is the minimal required time in months of formal pediatric training that family doctors/general practitioners undergo? | - None, 1, 1-3, 4-6, not applicable |
|  | 18. Who supervises training for GP/FD in primary pediatric care usually? | - GP/FD  - PCP  - Academic Pediatricians  - Trained Nurses  - not applicable |
| **Digitalization in Pediatrics** | 19. Are electronic health records maintained? | - Yes, No, Gradually introduced |
|  | 20. If yes or gradually introduced: please specify:  (multiple select) | - Patient records/medical history, Lab tests/ blood work, Imaging, e-Prescriptions, vaccinations, Well-Visit info like Milestones |
|  | 21. Who has access to these records(at least partially)?  (multiple select) | - Patient, PCP/GP  - Pharmacy(for prescriptions)  - Hospitals  - Multiple Access Points in Health System |
|  | 22. How is telemedicine (like digital communication tool or video consultations) used in your country?  (multiple select) | - for chronic consultations  - for acute consultations  - for Mental health |
