# Supplementary material for: Paediatric Primary Care Across Europe: A Survey of 42 Countries
Source: Acta Paediatr. 2025 Dec 4;115(4):821–31. doi: 10.1111/apa.70404 (PMC12975683; doi:10.1111/apa.70404)
Supplement: Supplementary file 8 — Table S2: Countries by geographical region according to standard country or area codes for statistical use by the UN. [file APA-115-821-s003.docx]

***Table S2*** *Countries by geographical region according to standard country or area codes for statistical use by the UN (M49)*

| Northern Europe | Western Europe | Eastern Europe | Southern Europe | Western Asia |
| --- | --- | --- | --- | --- |
| Denmark | Austria | Belarus | Albania | Armenia |
| Estonia | Belgium | Bulgaria | Bosnia and Herzegovina | Azerbaijan |
| Finland | France | Czech Republic | Croatia | Cyprus |
| Iceland | Germany | Hungary | Greece | Israel |
| Ireland | Luxembourg | Moldova | Italy | Türkiye |
| Lithuania | Netherlands | Poland | Malta |  |
| Norway | Switzerland | Romania | Montenegro |  |
| Sweden |  | Slovakia | North Macedonia |  |
| United Kingdom |  | Ukraine | Portugal |  |
|  |  |  | Serbia |  |
|  |  |  | Slovenia |  |
|  |  |  | Spain |  |
